# Serum matrix metalloproteinase‐7, Syndecan‐1, and CA 19‐9 as a biomarker panel for diagnosis of pancreatic ductal adenocarcinoma

**DOI:** 10.1002/cam4.70144

**Published:** 2024-09-12

**Authors:** Doron Yablecovitch, Moshe Nadler, Shomron Ben‐Horin, Orit Picard, Miri Yavzori, Ella Fudim, Moran Tardio Duchan, Emad Sakhnini, Alon Lang, Maor Lahav, Talia Saker, Sandra Neuman, Limor Selinger, Biana Freitz, Revital Dvir, Maria Raitses‐Gurevich, Talia Golan, Idan Levy, Ido Laish

**Affiliations:** ^1^ Gastroenterology Institute Chaim Sheba Medical Center Tel Hashomer Israel; ^2^ Sackler School of Medicine Tel Aviv University Tel Aviv Israel; ^3^ Shalvata Mental Health Center Hod Hasharon Israel; ^4^ Department of Oncology Chaim Sheba Medical Center Tel Hashomer Israel

**Keywords:** biomarkers, matrix metalloproteinase‐7, pancreatic cancer, Syndecan‐1

## Abstract

**Aims and Background:**

Matrix metalloproteinase‐7 (MMP‐7) and Syndecan‐1 (SDC1) are involved in multiple functions during tumorigenesis. We aimed to evaluate the diagnostic and prognostic performance of these serum proteins, as potential biomarkers, in patients with pancreatic ductal adenocarcinoma (PDAC) and benign pancreatic cysts.

**Methods:**

In this case–control study, patients with newly diagnosed PDAC (*N* = 121) were compared with the benign cyst (*N* = 66) and healthy control (*N* = 48) groups. Serum MMP‐7 and SDC1 were measured by ELISA. The diagnostic accuracy of their levels for diagnosing PDAC and pancreatic cysts was computed, and their association with survival outcomes was evaluated.

**Results:**

MMP‐7 median serum levels were significantly elevated in the PDAC (7.3 ng/mL) and cyst groups (3.7 ng/mL) compared with controls (2.9 ng/mL) (*p* < 0.001 and 0.02, respectively), and also between the PDAC and cyst groups (*p* < 0.001), while SDC1 median serum levels were significantly elevated in PDAC (43.3 ng/mL) compared with either cysts (30.1 ng/mL, *p* < 0.001) or controls (31.2 ng/mL, *p* < 0.001). The receiver operating characteristic curve analysis area under the curve in PDAC versus controls was 0.90 and 0.78 for MMP‐7 and SDC1, respectively, while it was 1.0 for the combination of the two and CA 19‐9 (*p* < 0.001). The combination of the three biomarkers had a perfect sensitivity (100%).

**Conclusions:**

Due to its high sensitivity, this biomarker panel has the potential to rule out PDAC in suspected cases.

## 1 INTRODUCTION

Pancreatic ductal adenocarcinoma (PDAC) is one of the leading causes of cancer death, with an estimated five‐year survival rate of only 13% despite the improvements in therapeutic strategies.^1^ Its poor prognosis relates not only to an intrinsic biological aggressiveness, but also to late presentation of clinical symptoms as well as lack of reliable strategies for early detection in average‐risk population.^2^ While most PDAC develop from Pancreatic Intraepithelial Neoplasia (PanIN), about 15%–20% grows from well‐defined pre‐malignant cystic lesions, namely intraductal papillary mucinous neoplasms (IPMNs) and mucinous cystic neoplasms (MCNs).^2^, ^3^ In the absence of biomarkers with sufficient diagnostic accuracy, current pancreas neoplasia screening relies on imaging or endoscopic ultrasonography (EUS) to differentiate between low‐risk and high‐risk lesions for developing PDAC.^4^

Matrix metalloproteinases (MMPs) comprise a family of at least 25 secreted and cell surface zinc‐dependent endopeptidases that are involved in physiological and pathological remodeling of extracellular matrix,^5^ but also in regulating signaling pathways that control cell growth, and as such are also involved in tumor progression and invasion of surrounding tissue.^6^, ^7^ Specifically, MMP‐7 is overexpressed in PanIN and was shown to be upregulated approximately 10‐fold in pancreatic cancer.^8^ Furthermore, abundant MMP‐7 expression promotes the proliferation, invasion, and migration of tumor cells and was indeed associated with lower survival rates in PDAC patients.^9^, ^10^, ^11^ Plasma concentrations of MMP‐7 were also shown to be higher in PDAC and associated with poor prognosis.^11^, ^12^, ^13^

Syndecan‐1 (SDC1) is a member of the transmembrane heparan sulfate proteoglycan family and is one the proteins that are released by MMP‐7 to the serum. Although primarily involved in cell adhesion, migration, and cell‐matrix interactions,^14^, ^15^ it also regulates multiple functions during tumorigenesis, including tumor cell attachment, proliferation, and angiogenesis through different signaling pathways (e.g., Wnt pathway activation).^16^, ^17^ In the era of PDAC, two recent published studies have linked SDC1 levels with mutated overexpressed KRAS, the initiating step in most PDACs, which cooperate to induce a malignant phenotype through regulation of macropinocytosis, a critical metabolic pathway that fuels PDAC cell growth and promotes tumor progression.^18^, ^19^ Moreover, we have recently shown elevated baseline serum SDC1 levels in PDAC compared to healthy individuals,^20^ potentially serving as a diagnostic biomarker.

Although these two proteins were previously evaluated as potential biomarkers in PDAC, they have not been hitherto evaluated in combination in PDAC or in pre‐malignant cystic lesions, neither was their combination with CA 19‐9 explored for a potential increased diagnostic accuracy. We thus aimed to evaluate the diagnostic and prognostic performance of serum MMP‐7 and SDC1 as potential biomarkers, separately and combined, and as adjunct to CA 19‐9, in patients with PDAC and pancreatic cysts.

## 2 METHODS

### 2.1 Design and patient population

This was a case–control study conducted at the Sheba Medical Center, a tertiary academic center in Israel. We included two prospectively recruited study populations: (1) Patients with newly diagnosed PDAC by either EUS‐guided biopsy or by surgically obtained specimen. (2) Patients with newly diagnosed pancreatic cysts, based on either EUS evaluation or surgical resection, coupled with subsequent follow‐up history. The serum samples from all patients were obtained from two sources: (1) The Sheba Medical Center's Tissue Bank Repository for PDAC patients and those with pancreatic cysts who underwent upfront surgery between September 2014 and December 2022; (2) The Gastrointestinal (GI) Institute for patients with newly diagnosed EUS‐ based disease between October 2019 and December 2022. Healthy controls were recruited after visiting the GI clinics for general symptoms. All patients were followed up through December 2022.

In all patients, baseline serum markers samples obtained before any surgical or oncological intervention were tested for the tumor marker CA 19‐9, SDC1, and MMP‐7. The data collected from medical files included demographic characteristics, presence of diabetes, smoking habits, tumor/cyst location, germline testing (if performed), cyst characteristics, pathological/clinical staging, surgery conduction, and date of censor (December 2022)/death. Exclusion criteria from the study included patients who were unable to sign an informed consent (see below), patients who suffered from autoimmune disorders, systemic infections, or extra‐pancreatic malignancies. All patients provided an informed consent (either for this study or for the central tissue banking), Sheba institutional ethics review board approved the study (SMC‐6185‐19).

### 2.2 Definitions and classifications

PDAC staging was classified either by three clinically distinct patient groups: resectable (T1‐3, stages 1 and 2), locally advanced (T4, stage 3), and metastatic disease (M1, stage 4), or by the American Joint Committee on Cancer (AJCC)/TMN system staging for PDAC. Determination of staging was based on either pathological assessment for resectable ones or imaging modalities (mainly CT scans) for non‐resectable tumors. We considered preoperative staging for the purpose of categorization for locally advanced/borderline tumors in which neo‐adjuvant treatment was given before surgery.

For cystic lesions, worrisome features and high‐risk stigmata for IPMN were defined in this study according to the Sendai and Fukuoka International Consensus Guidelines for management of mucinous cysts.^4^ For surgically removed lesions, high‐grade precursor lesions were defined as PanIN‐3 /IPMN with high‐grade dysplasia. All other lesions (namely, benign cystic lesions with no high‐risk stigmata, or low‐grade precursor lesions like PanIN‐1, PanIN‐2, and IPMN with low‐or moderate‐grade dysplasia) were considered non‐significant.

### 2.3 Soluble Syndecan‐1 analysis

Serum samples were collected, centrifuged at 3000 rpm for 10 min and stored at −80°C. Serum SDC1 levels were evaluated using human SDC1 ELISA (Diaclone Research, Besancon, France) according to the instructions provided by the manufacturer. The standards and samples were analyzed in duplicate. Concentrations of Serum SDC1 were reported as ng/mL. The technicians were blinded to the clinical data.

### 2.4 Serum matrix metalloproteinase‐7analysis

Serum samples were collected, centrifuged at 3000 rpm for 10 min and stored at −80°C. Concentrations of serum MMP‐7 were evaluated using human MMP‐7 ELISA (R&D systems, Minneapolis, MN, USA) following the manufacturer's instructions, using standards provided with the kit. Standards and samples were analyzed in duplicate. Serum MMP‐7 levels were reported as ng/mL. The technicians were blinded to the clinical data.

### 2.5 CA 19‐9 analysis

Serum CA 19‐9 was quantitatively determined by the Access® GI Monitor assay on the Beckman Coulter Unicell DXI 800 following the clinical and laboratory standards institute guidelines.

### 2.6 Statistical analysis

Categorical variables were summarized as frequencies and percentages. The distribution of continuous variables was evaluated using histograms. Since all continuous variables were not normally distributed, they were reported as median and interquartile range (IQR). The Mann–Whitney *U*‐test was used to compare the continuous variables between groups and chi‐squared test was used to compare the categorical variables between the two groups. The area under the Receiver operating characteristic (ROC) curve was used to evaluate the ability of the biomarkers to discriminate between the patient groups. The areas under the ROC curve were compared using DeLong test. Logistic regression was used to evaluate the patients' probabilities of having cancer based on biomarkers. Chi‐squared automatic interaction detection (KASS 1980) was used to identify subgroups of patients. Spearman's correlation coefficient was used to study the correlation between the biomarkers. Log‐rank test and Kaplan–Meier curve were used to compare the association between the biomarkers categories and patients' survival. All statistical tests were 2‐sided, and *p* < 0.05 was considered statistically significant. All statistical analyses were performed using SPSS version 28 (IBM, Armonk, NY, USA).

## 3 RESULTS

### 3.1 Patient characteristics

The characteristics of the study population are depicted in Table 1. The median (IQR) age was 70 (64–75), 70 (65–75), and 67 (59–73) for the PDAC (*N* = 121), cyst (*N* = 66), and control (*N* = 48) groups (*p* = 0.08), respectively. The rates of diabetes and smoking were significantly higher in the PDAC group than in the cyst and control groups, and male gender was more frequent among the PDAC group (*p* = 0.06).

**TABLE 1:** Demographic and clinical characteristics of the study groups.

	PDAC (*N* = 121), *N* (%)	Cyst (*N* = 66), *N* (%)	Control (*N* = 48), *N* (%)	*p* value
Age, median (IQR)	70 (64–75)	70 (65–75)	67 (59–73)	0.08
Male gender	65 (53.7)	26 (39.4)	21 (43.8)	0.06
Smoking habits	36 (29.8)	7 (10.6)	3 (6.3)	0.001^a^, 0.003^b^
Diabetes	53 (43.8)	15 (22.7)	12 (25.0)	0.023^a^, 0.04^b^
Clinical staging				
Resectable	21 (17.4)			
Locally advanced	60 (49.6)			
Metastatic	40 (33.0)			
Endoscopic features of cysts				
No worrisome features		49 (74.2)		
Size >30 mm		8 (12.1)		
MPD 5–9 mm		5 (7.6)		
MPD ≥10 mm		4 (6.1)		
Pathologic features of resected cysts				
LGD or no dysplasia		8 (12.1)		
HGD		6 (9.0)		
CA 19‐9 >37 U/mL	100 (82.6)	0	0	<0.001^a^, ^b^

^a^ Between the PDAC and control groups.

^b^ Between the PDAC and cyst groups.

Abbreviation: PDAC, pancreatic ductal adenocarcinoma.

Among PDAC patients, 21 (17.4%) had resectable tumors, 60 (49.6%) had locally advanced disease, and 40 (33.0%) were metastatic. The tumor was localized to the head, body, and tail in 82 (67.8%), 25 (20.6%), and 14 (11.6%) of patents, respectively. Among cyst patients, 60 (90.9%) had IPMN and 6 (9.1) had mucinous cysts. Seventeen (25.7%) patients had cysts with either endoscopically worrisome features (cyst size >3 cm or main pancreatic duct—MPD diameter 5–9 mm) or high‐risk stigmata (MPD diameter >10 mm). There were no cases of enhancing mural nodule. Of surgically removed lesions (*N* = 14), six harbored high‐grade dysplasia and the rest had low grade or no dysplasia.

### 3.2 PDAC versus control groups

**FIGURE 1:**
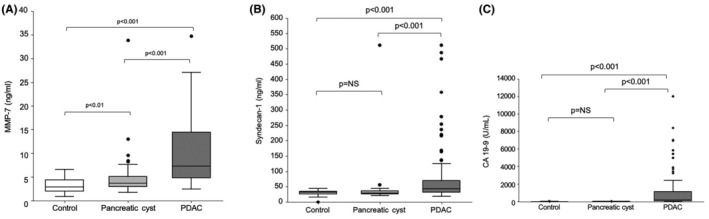
(A) Box‐plot representation of median serum matrix metalloproteinase‐7 levels (ng/mL) in patients with pancreatic ductal adenocarcinoma (PDAC) versus pancreatic cyst versus healthy controls. (B) Box‐plot representation of median serum Syndecan‐1 levels (ng/mL) in patients with PDAC versus pancreatic cysts versus healthy controls. (C) Box‐plot representation of median CA 19‐9 levels (U/mL) in patients with PDAC versus pancreatic cysts versus healthy controls.

**FIGURE 2:**
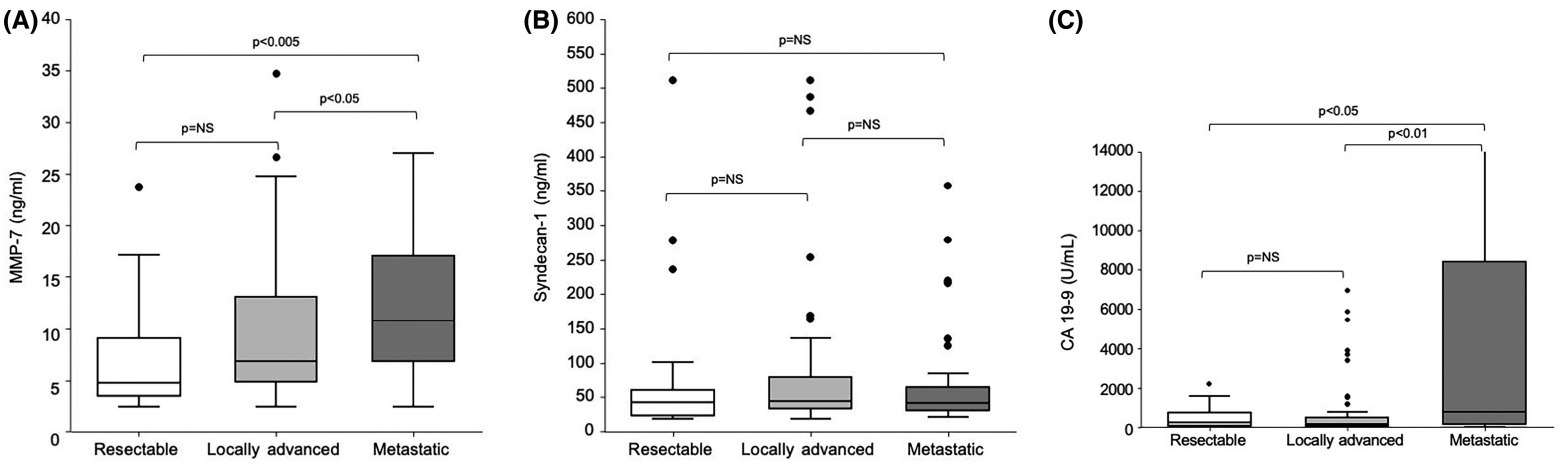
(A) Box‐plot representation of median serum matrix metalloproteinase‐7 levels (ng/mL) in patients with pancreatic ductal adenocarcinoma (PDAC) according to disease progression. (B) Box‐plot representation of median serum Syndecan‐1 levels (ng/mL) in patients with PDAC according to disease progression. (C) Box‐plot representation of median CA 19‐9 levels (U/mL) in patients with PDAC versus pancreatic cysts versus healthy controls.

MMP‐7 median serum levels were significantly elevated in PDAC compared with controls (7.3 ng/mL [IQR 4.8–14.5] vs. 2.9 ng/mL, [IQR 2.0–4.4], respectively; *p* < 0.001) (Figure 1A), and median serum level of SDC1 were similarly elevated in PDAC versus controls (43.3 ng/mL [IQR 32.2–71.2] vs. 31.2 ng/mL [IQR 26.3–35.1], respectively; *p* < 0.001) (Figure 1B). Levels of CA 19‐9 were also higher in PDAC compared with controls (239.0 U/mL [IQR 75–1137] vs. 8.0 U/mL [IQR 4.0–12.2], respectively; *p* < 0.001) (Figure 1C). Within the PDAC group, MMP‐7 and CA 19‐9 levels were significantly elevated with disease progression (Figure 2A,C), while SDC1 levels were not associated with PDAC stage (Figure 2B).

**FIGURE 3:**
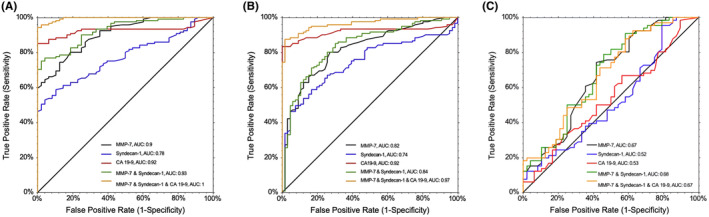
(A) Receiver operating characteristic curve (ROC) of serum Syndecan‐1, serum matrix metalloproteinase‐7 (MMP‐7), and CA 19‐9, in single and combined, for diagnosis of pancreatic ductal adenocarcinoma (PDAC) versus healthy controls. (B) ROC curve of serum Syndecan‐1, serum MMP‐7, and CA 19‐9, in single and combined, for diagnosis of PDAC versus pancreatic cyst. (C) ROC curve of serum Syndecan‐1, serum MMP‐7, and CA 19‐9, single and combined, for diagnosis of pancreatic cysts versus healthy controls.

According to ROC analysis, the area under the curve (AUC) was 0.90 (confidence interval [CI] 0.86–0.95; *p* < 0.001) for MMP‐7, 0.78 (CI 0.70–0.84; *p* < 0.001) for SDC1 and 0.92 (CI 0.88–0.96; *p* < 0.001) for CA 19‐9 (used as a continuous variable), between the PDAC and control groups, while the combination of the three biomarkers increased the AUC to 1.0 (CI 0.99–1.0; *p* < 0.001) (Figure 3A and Table 2).

**TABLE 2:** Diagnostic accuracies of the serum biomarkers for PDAC versus control populations.

Biomarker	Sensitivity (%)	Specificity (%)	LR+	LR−	AUC (95% CI)
Syndecan‐1	83	46	1.54	0.37	0.78 (0.70–0.84)
MMP‐7	93	65	2.66	0.11	0.9 (0.86–0.95)
CA 19‐9	83	100	NA	0.17	0.92 (0.88–0.96)
Syndecan‐1 and MMP‐7	98	33	1.46	0.06	0.93 (0.90–0.95)
Syndecan‐1 and CA 19‐9	97	46	1.80	0.07	0.97 (0.94–0.99)
MMP‐7 and CA 19‐9	99	65	2.83	0.02	0.99 (0.98–1.0)
Syndecan‐1 and MMP‐7 and CA 19‐9	100	33	1.49	0.00	1.0 (0.99–1.0)

*Note*: Sensitivity, specificity, LR+ and LR− were calculated using the following cut‐off values: Syndecan‐1: 30 ng/mL, MMP‐7: 3.5 ng/mL and CA 19‐9: 37 U/mL.

Abbreviations: LR+, positive likelihood ratio; LR−, negative likelihood ration; PDAC, pancreatic ductal adenocarcinoma.

Using ROC analysis for PDAC, a cutoff level of 3.5 ng/mL serum MMP‐7 showed a sensitivity of 92% and a specificity of 65%, while cutoff level of 30 ng/mL serum SDC1 showed a sensitivity of 83% and a specificity of 46% (Table 2). Notably, using CA 19‐9 as a dichotomic variable according to standard laboratory cutoff level, a subset of PDAC patients with normal CA 19‐9 (<37 U/mL) had elevated serum level of MMP‐7 and SDC1. Of 21 (17.3%) PDAC patients with normal serum CA 19‐9, 5 (23.8%) had metastatic disease and the rest had stage 1–2 disease. Using the above‐mentioned cutoffs, 20/21 (95.2%) and 18/21 (85.7%) of these patients had positive MMP‐7 and SDC1 levels, respectively. The single patient with both negative CA 19‐9 and MMP‐7 levels, was positive for SDC1. The diagnostic accuracies of the biomarkers are presented in Table 2.

The correlation between MMP‐7 and SDC1 and between MMP‐7 and CA 19‐9 were both weak, despite being statistically significant (*r* = 0.25, *p* = 0.006; *r* = 0.20, *p* = 0.001, respectively).

### 3.3 PDAC versus cyst groups

MMP‐7 median serum levels were significantly elevated in the PDAC group compared with the cyst group (7.3 ng/mL [IQR 4.8–14.5] vs. 3.7 ng/mL [IQR 2.9–5.0], respectively; *p* < 0.001) (Figure 1A). This was also true for median serum level of SDC1 which were elevated in PDAC compared with pancreatic cysts patients (43.3 ng/mL [IQR 32.2–71.2] vs. 30.1 ng/mL [IQR 26.5–35.4], respectively; *p* < 0.001) (Figure 1B). Levels of CA 19‐9 were also higher in PDAC compared with cysts (239.0 U/mL [IQR 75–1137] vs. 8.5 U/mL [IQR 4.0–14.1], respectively; *p* < 0.001) (Figure 1C). Within the cyst group, there were no differences in the levels of the three markers between cysts with high‐risk endoscopic stigmata or harboring HGD and those without. Upon ROC analysis, the AUC was 0.82 (CI 0.75–0.88; *p* < 0.001) for MMP‐7 and 0.74 (CI 0.67–0.81; *p* < 0.001) for SDC1, while their combination non‐significantly increased the AUC to 0.84 (CI 0.72–0.89; *p* = 0.18) (Figure 3B). The diagnostic accuracies of the biomarkers are presented in Table 3.

**TABLE 3:** Diagnostic accuracies of the serum biomarkers for PDAC versus cyst populations.

Biomarker	Sensitivity (%)	Specificity (%)	LR+	LR−	AUC (95% CI)
Syndecan‐1	83	48	1.60	0.35	0.74 (0.67–0.81)
MMP‐7	93	35	1.43	0.20	0.82 (0.75–0.88)
CA 19‐9	83	100	NA	0.17	0.92 (0.88–0.96)
Syndecan‐1 and MMP‐7	98	17	1.18	0.12	0.84 (0.72–0.89)
Syndecan‐1 and CA 19‐9	97	48	1.87	0.06	0.93 (0.89–0.97)
MMP‐7 and CA 19‐9	99	35	1.52	0.03	0.97 (0.94–0.99)
Syndecan‐1 and MMP‐7 and CA 19‐9	100	17	1.20	0.00	0.97 (0.94–0.99)

*Note*: Sensitivity, specificity, LR+ and LR– were calculated using the following cut‐off values: Syndecan‐1: 30 ng/mL, MMP‐7: 3.5 ng/mL and CA 19‐9: 37 U/mL.

Abbreviations: LR+, positive likelihood ratio; LR−, negative likelihood ration; PDAC, pancreatic ductal adenocarcinoma.

### 3.4 Cyst versus control groups

MMP‐7 median serum level was significantly elevated in the cyst group compared with controls (3.7 ng/mL [IQR 2.9–5.0] vs. 2.9 ng/mL [IQR 2.0–4.4]; *p* = 0.02, respectively) (Figure 1A), while SDC1 median serum level was not significantly different between the two groups (30.1 ng/mL [IQR 26.5–35.4] vs. 31.2 ng/mL [IQR 26.3–35.1]; *p* = 0.874, respectively) (Figure 1B). Levels of CA 19‐9 were also comparable between the two groups (*p* = 0.68) (Figure 1C). None of the patients in either group had elevated (>37 U/mL) serum level of CA 19‐9. Upon ROC analysis, the AUC was 0.67 (CI 0.56–0.77; *p* = 0.002) for MMP‐7 but only 0.52 (CI 0.41–0.63; *p* = 0.67) for SDC1, and the combination of the two serum markers did not increase the AUC >0.67 (Figure 3C).

### 3.5 Survival prediction within the PDAC group

**FIGURE 4:**
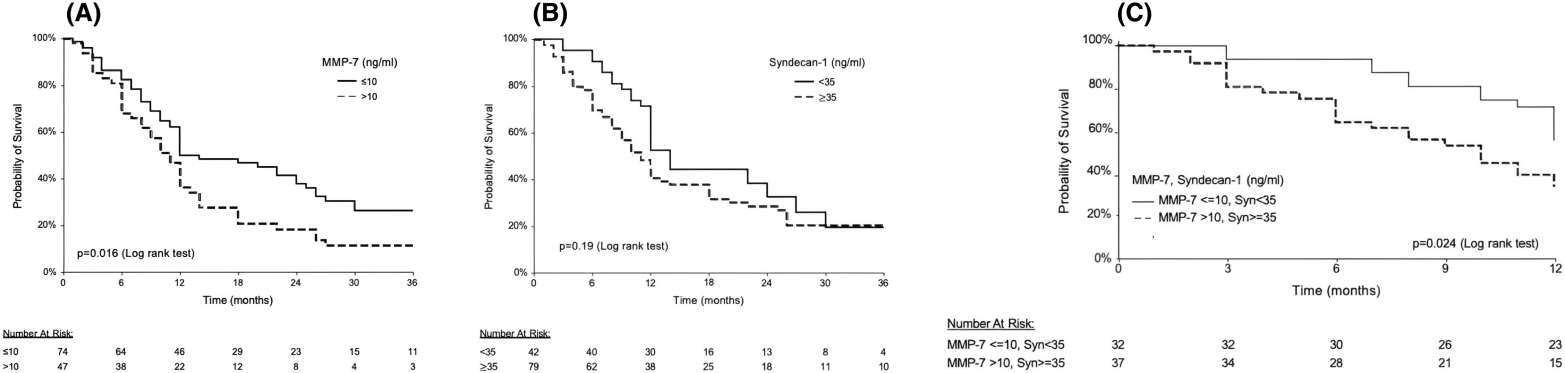
(A) Kaplan–Meier curve of overall 3‐year survival among patients with serum matrix metalloproteinase‐7 (MMP‐7) levels >10 ng/mL at baseline versus those with baseline serum MMP‐7 levels ≤10 ng/mL. (B) Kaplan–Meier curve of overall 3‐year survival among patients with serum Syndecan‐1 levels ≥35 ng/mL at baseline versus those with baseline serum Syndecan‐1 levels <35 ng/m. (C) Kaplan–Meier curve of overall 1‐year survival among patients with combined serum Syndecan‐1 levels ≥35 ng/mL at baseline and baseline serum MMP‐7 levels >10 ng/mL versus those with lower combined levels.

Overall, 31/121 (25.6%) of the patients were alive at the time of censoring, with a follow‐up time between 12 and 110 months (median—12 months, IQR 6–20). For patients with baseline serum MMP‐7 ≤10 ng/mL versus >10 ng/mL, there was a trend for survival benefit after 1 year (50% vs. 36%, respectively; *p* = 0.095) and a significant benefit after 3 years (14% vs. 7%, respectively; *p* = 0.016) (Figure 4A). For patients with baseline serum SDC1 <35 ng/mL versus ≥35 ng/mL, there was a trend for survival benefit after 1 year (52% vs. 40%, respectively; *p* = 0.06) and no benefit after 3 years (19% vs. 20%, respectively; *p* = 0.193) (Figure 4B). However, patients with high serum levels of both SDC1 (≥35 ng/mL) and MMP‐7 (>10 ng/mL) had higher mortality rate after 1 year compared with patients with lower serum levels of both markers (56% vs. 35%, respectively; *p* = 0.024) (Figure 4C).

## 4 DISCUSSION

In this study, we found that a combination of three biomarkers, namely serum MMP‐7, SDC1, and CA 19‐9, provided superior diagnostic accuracy for PDAC compared with any of these biomarkers alone, with a perfect sensitivity to PDAC. Moreover, MMP‐7 levels correlated with more advanced, metastatic disease and the combination of MMP‐7 and SDC1 demonstrated prognostic value in PDAC.

MMP‐7 was previously assessed in several studies and found to be associated with metastatic PDAC. Its tissue levels were positively correlated with the severity of all categories of pathological staging and associated with two to threefold shorter mean survival times in several patient cohorts.^8^, ^9^, ^10^, ^11^ Serum levels were also assessed for their utility for predicting severity of disease and survival. In one surgical study, MMP‐7 had modest utility to predict unresectability and nodal involvement (AUC = 0.68), while for patients with levels above 13.5 ng/mL (approximately 15% of the cohort), it was highly predictive for unresectable disease.^12^ In another study, all PDAC patients without lymph node involvement had MMP‐7 serum levels <20 ng/mL, versus levels >20 ng/mL in all metastatic patients.^11^ Moreover, the combined monitoring of serum MMP‐7 and CA 19‐9 improved the predictive value for PDAC compared with MMP‐7 alone, yielding AUC of 0.90–0.99.^13^, ^21^ In comparison to these studies, our results show AUC of 0.90 and 0.99 for MMP‐7 exclusively and in combination with CA 19‐9, respectively. In addition to its diagnostic utility, MMP‐7, separately and combined with SDC1, had prognostic value in PDAC. Notably, the cutoff of MMP‐7 for its diagnostic and prognostic values differed, being 3.5 and 10 ng/mL, respectively.

In contrary to MMP‐7, there is limited data of the role of SDC1 in PDAC. SDC1 was first shown to be over‐expressed in pancreatic tissues from patients with progression to PDAC.^22^, ^23^ However, two recent studies have linked its pathogenic role to mutated KRAS,^18^, ^19^ the initiating step in the majority of PDACs, which contributes to the induction of malignant phenotype. In a landmark study by Yao and colleagues, in transgenic mice models of PDAC, oncogenic KRAS induced SDC1 cell surface overexpression, where it regulated macropinocytosis, a crucial metabolic pathway that fuels PDAC cell growth and promotes tumor progression.^18^ Recently, Zhang et al. found that the expression of SDC1 indicated a poor survival in PDAC patients and confirmed that depletion of SDC1 can significantly suppress in‐vitro PDAC cell proliferation, induce cell apoptosis, and impair cell migration.^24^

Thus, the combined use of MMP‐7 and SDC1 has pathophysiologic rationale. MMP‐7 was demonstrated to shed SDC1 complexes with CXC chemokine from cell surfaces, where it was established in spatially localizing neutrophil activation to epithelia.^25^, ^26^ Since the MMP‐7‐induced shedding of SDC1 ectodomain was previously observed in cultured pancreatic cancer cell lines,^27^ it is possible that the increased serum SDC1 level in our cohort was related to MMP‐7. Indeed, in our study we observed a correlation between serum levels of these two proteins, as expected. However, the superior sensitivity and yield of serum MMP‐7 levels over SDC1 in PDAC, suggests that it may be the dominant modulator in tumor progression and microenvironment, possibly thorough several mechanisms of which one is the release of SDC1 ectodomain. Our observation that the combined use of MMP‐7 and SDC1 modestly increased diagnostic sensitivity for PDAC and that some patients had only elevated serum SDC1 level, may suggest additional mechanisms for its shedding other than cleavage by MMP. These may include tumor‐induced overexpression of cell surface SDC1^28^ or upregulation of heparanase, an endoglycosidase that specifically degrades the heparan‐sulfate chains of SDC1.^29^, ^30^ The potential effect of MMP‐7‐induced shedding of SDC1 on macropinocytosis in PDAC remains to be clarified.

The combined three‐biomarkers panel showed the potential optimal diagnostic yield for PDAC. Although shown in a small cohort, a sensitivity of 100% could make it an ideal panel for ruling out PDAC in suspected cases. However, in light of the fact that most of our cohort (>80%) had advanced or metastatic disease, the utility for early detection of the panel could not be assessed. Nonetheless, additional diagnostic tools, including imaging and blood‐based biomarkers, are being developed and we hypothesize that a combination panel (blood based and imaging) will perform the best strategy for early detection. Notably, despite an isolated specificity of 100% for CA 19‐9 in our study, probably affected by the relatively small study sample of healthy individuals, previous studies showed a more limited diagnostic accuracy as well, with PPV between 80% and 90%.^31^

Our study is the first to show that benign cystic pancreatic lesions were associated with significantly higher serum levels of MMP‐7 than controls. In contrast, serum levels were not different between low‐ versus high‐risk cystic lesions, although this observation must be interpreted cautiously, considering the small patient number with high‐risk features. Importantly, other benign pre‐malignant pancreatic lesions, for example, chronic pancreatitis (CP) and benign peri‐pancreatic neoplasms (duodenal/papillary adenomas), were not associated with high serum levels of MMP‐7.^13^ However, upregulated tissue expression was demonstrated in patients with these pre‐malignant pancreatic lesions (CP and metaplastic duct lesions).^8^ Although cystic MMP‐7 was not measured in our study, its high serum level thus supports the existence of upregulated tissue expression within pre‐malignant cysts with spillage to the serum. SDC1, notably was one of the differentially expressed genes identified in IPMN lesions.^24^ The observation that it was not translated to significantly elevated serum levels in our study may be due to several possible factors, such as low level protein production by pre‐cancerous cells, low level production of cells that constitutively express SDC1 that have been shed into the circulation or differential access to the circulation. Further studies should elucidate this point as well whether other pre‐cancerous lesions like mucinous cysts and PanIN, do express MMP‐7 and SDC1.

The limitations to our study include serum MMP‐7 and SDC1 were measured in a single time‐point before any treatment, which limits our ability to study its prognostic role and its correlation to treatment. Also, we did not check for Lewis antigen status in our cohort, which may limit the diagnostic yield of CA 19‐9. Another limitation was the lack of zymography in MMP‐7 analysis of the serum samples. Using ELISA, these molecules are measured in totality, whereas, zymography may reveal, individually, the activated forms and degradation products of MMP‐7. A small cohort of patients with high‐grade cysts prevented us from showing a potential grading of serum level within this group. Finally, extrapolation to more heterogeneous populations need to be shown, since our cohort is from a relatively homogenous Israeli population.

In conclusion, this study shows the potential diagnostic use of a combination of serum biomarkers, consisting of MMP‐7, SDC1, and CA 19‐9, for the detection of PDAC, as all patients expressed one of these proteins in excess in their blood. Moreover, serum MMP‐7 was modestly elevated in benign cystic lesions, although no difference was shown between low‐ and high‐grade lesions, limiting its utility in this setting. However, more large‐scale, and prospective studies should be performed to assess MMP‐7 and SDC1, exact pathogenetic role in the development of these lesions and whether it can differentiate between simple and more high‐risk lesions, potentially needing closer follow‐up or surgery. Lastly, these combinatorial markers may be of value, as diagnostic biomarkers in carriers with high‐risk potential to develop PDAC, for example, BRCA carriership or Peutz–Jehger. Future studies on high‐risk groups in international consortium would be a preferential platform to explore this biomarker panel.

## CONFLICT OF INTEREST STATEMENT

The authors declare no conflict of interest.

## Data Availability

The data that support the findings of this study are available from the corresponding author upon reasonable request.

## References

[1] Siegel RL , Miller KD , Jemal A . Cancer statistics, 2019. CA Cancer J Clin. 2019;69:7‐34. 30620402 10.3322/caac.21551

[2] Kleeff J , Korc M , Apte M , et al. Pancreatic cancer. Nat Rev Dis Primers. 2016;2:16022. 27158978 10.1038/nrdp.2016.22

[3] Ryan DP , Hong TS , Bardeesy N . Pancreatic adenocarcinoma. NEngl J Med. 2014;371:1039‐1049. 10.1056/NEJMra1404198 25207767

[4] Tanaka M , Fernandez‐del Castillo C , Adsay V , et al. International consensus guidelines 2012 for the management of IPMN and MCN of the pancreas. Pancreatology. 2012;12:183‐197. 22687371 10.1016/j.pan.2012.04.004

[5] Cui N , Hu M , Khalil RA . Biochemical and biological attributes of matrix metalloproteinases. Prog Mol Biol Transl Sci. 2017;147:1‐73. 28413025 10.1016/bs.pmbts.2017.02.005 PMC5430303

[6] Gonzalez‐Avila G , Sommer B , Mendoza‐Posada DA , Ramos C , Garcia‐Hernandez AA , Falfan‐Valencia R . Matrix metalloproteinases participation in the metastatic process and their diagnostic and therapeutic applications in cancer. Crit Rev Oncol Hematol. 2019;137:57‐83. 31014516 10.1016/j.critrevonc.2019.02.010

[7] Egeblad M , Werb Z . New functions for the matrix metalloproteinases in cancer progression. Nat Rev Cancer. 2002;2:161‐174. 11990853 10.1038/nrc745

[8] Crawford HC , Scoggins CR , Washington MK , Matrisian LM , Leach SD . Matrix metalloproteinase‐7 is expressed by pancreatic cancer precursors and regulates acinar‐to‐ductal metaplasia in exocrine pancreas. J Clin Invest. 2002;109:1437‐1444. 12045257 10.1172/JCI15051 PMC150998

[9] Yamamoto H , Itoh F , Iku S , et al. Expression of matrix metalloproteinases and tissue inhibitors of metalloproteinases in human pancreatic adenocarcinomas: clinicopathologic and prognostic significance of matrilysin expression. J Clin Oncol. 2001;19:1118‐1127. 11181677 10.1200/JCO.2001.19.4.1118

[10] Fukushima H , Yamamoto H , Itoh F , et al. Association of matrilysin mRNA expression with K‐ras mutations and progression in pancreatic ductal adenocarcinomas. Carcinogenesis. 2001;22:1049‐1052. 11408348 10.1093/carcin/22.7.1049

[11] Fukuda A , Wang SC , Morris JP 4th , et al. Stat3 and MMP7 contribute to pancreatic ductal adenocarcinoma initiation and progression. Cancer Cell. 2011;19:441‐455. 21481787 10.1016/j.ccr.2011.03.002 PMC3075548

[12] Wang SC , Parekh JR , Porembka MR , et al. A pilot study evaluating serum MMP7 as a preoperative prognostic marker for pancreatic ductal adenocarcinoma patients. J Gastrointest Surg. 2016;20:899‐904. 26921028 10.1007/s11605-015-3057-z PMC4851562

[13] Kuhlmann KFD , van Till JWO , Boermeester MA , et al. Evaluation of matrix metalloproteinase 7 in plasma and pancreatic juice as a biomarker for pancreatic cancer. Cancer Epidemiol Biomarkers Prev. 2007;16:886‐891. 17507610 10.1158/1055-9965.EPI-06-0779 PMC4516164

[14] Bartlett AH , Hayashida K , Park PW . Molecular and cellular mechanisms of syndecans in tissue injury and inflammation. Mol Cells. 2007;24:153‐166. 17978567

[15] Afratis NA , Nikitovic D , Multhaupt HB , Theocharis AD , Couchman JR , Karamanos NK . Syndecans—key regulators of cell signaling and biological functions. FEBS J. 2017;284(1):27‐41. 27790852 10.1111/febs.13940

[16] Teng YH , Aquino RS , Park PW . Molecular functions of Syndecan‐1 in disease. Matrix Biol. 2012;31:3‐16. 22033227 10.1016/j.matbio.2011.10.001 PMC3568394

[17] Czarnowski D . Syndecans in cancer: a review of function, expression, prognostic value, and therapeutic significance. Cancer Treat Res Commun. 2021;27:100312. 33485180 10.1016/j.ctarc.2021.100312

[18] Yao W , Rose JL , Wang W , et al. Syndecan 1 is a critical mediator of macropinocytosis in pancreatic cancer. Nature. 2019;568:410‐414. 30918400 10.1038/s41586-019-1062-1 PMC6661074

[19] Wu Y , Huang H , Fervers B , Lu L . Syndecan‐1 and KRAS gene expression signature associates with patient survival in pancreatic cancer. Pancreas. 2020;49:1187‐1194. 32898003 10.1097/MPA.0000000000001654

[20] Yablecovitch D , Ben‐Horin S , Picard O , et al. Serum Syndecan‐1: a novel biomarker for pancreatic ductal adenocarcinoma. Clin Transl Gastroenterol. 2022;13(5):e00473. 35297817 10.14309/ctg.0000000000000473 PMC9132524

[21] Resovi A , Bani MR , Porcu L , et al. Soluble stroma‐related biomarkers of pancreatic cancer. EMBO Mol Med. 2018;10:e8741. 29941541 10.15252/emmm.201708741 PMC6079536

[22] Juuti A , Nordling S , Lundin J , Louhimo J , Haglund C . Syndecan‐1 expression–a novel prognostic marker in pancreatic cancer. Oncology. 2005;68:97‐106. 15886501 10.1159/000085702

[23] Conejo JR , Kleeff J , Koliopanos A , et al. Syndecan‐1 expression is up‐regulated in pancreatic but not in other gastrointestinal cancers. Int J Cancer. 2000;88:12‐20. 10962434 10.1002/1097-0215(20001001)88:1<12::aid-ijc3>3.0.co;2-t

[24] Zhang CL , Shen Q , Liu FD , et al. SDC1 and ITGA2 as novel prognostic biomarkers for PDAC related to IPMN. Sci Rep. 2023;13(1):18727. 37907515 10.1038/s41598-023-44646-x PMC10618477

[25] Li Q , Park PW , Wilson CL , Parks WC . Matrilysin shedding of Syndecan‐1 regulates chemokine mobilization and transepithelial efflux of neutrophils in acute lung injury. Cell. 2002;111:635‐646. 12464176 10.1016/s0092-8674(02)01079-6

[26] Swee M , Wilson CL , Wang Y , McGuire JK , Parks WC . Matrix metalloproteinase‐7 (matrilysin) controls neutrophil egress by generating chemokine gradients. J Leukoc Biol. 2008;83:1404‐1412. 18334539 10.1189/jlb.0108016 PMC4139120

[27] Ding K , Lopez‐Burks M , Sanchez‐Duran JA , et al. Growth factor induced shedding of Syndecan‐1 confers Glypican‐1 dependence on mitogenic responses of cancer cells. J Cell Biol. 2005;171:729‐738. 16286510 10.1083/jcb.200508010 PMC2171561

[28] Malek‐Hosseini Z , Jelodar S , Talei A , Ghaderi A , Doroudchi M . Elevated Syndecan‐1 levels in the sera of patients with breast cancer correlate with tumor size. Breast Cancer. 2017;24(6):742‐747. 28382590 10.1007/s12282-017-0773-0

[29] Yang Y , MacLeod V , Miao HQ , et al. Heparanase enhances Syndecan‐1 shedding: a novel mechanism for stimulation of tumor growth and metastasis. J BiolChem. 2007;282:13326‐13333. 10.1074/jbc.M611259200 17347152

[30] Chen X , Zhao H , Chen C , et al. The HPA/SDC1 axis promotes invasion and metastasis of pancreatic cancer cells by activating EMT via FGF2 upregulation. Oncol Lett. 2020;19:211‐220. 31897132 10.3892/ol.2019.11121 PMC6924090

[31] Ballehaninna UK , Chambwrlain RS . The clinical utility of serum CA 19‐9 in the diagnosis, prognosis and management of pancreatic adenocarcinoma: an evidence‐based appraisal. J Gastrointest Oncol. 2012;3:105‐119. 22811878 10.3978/j.issn.2078-6891.2011.021 PMC3397644

